# Der neue Finanzausgleich zwischen Bund und Ländern: erste Ergebnisse und Bewertungen

**DOI:** 10.1007/s10273-021-2966-6

**Published:** 2021-07-19

**Authors:** André W. Heinemann

**Affiliations:** FB Wirtschaftswissenschaft, Universität Bremen, Wilhelm-Herbst-Straße 12, 28359 Bremen, Deutschland

**Keywords:** H24, H22, D31

## Abstract

Bereits 2015 wurde erstmalig durch die Konferenz der Ministerpräsident:innen ein neues Finanzausgleichssystem ab 2020 diskutiert und beschlossen. Seitdem sind zahlreiche Beiträge zu den möglichen fiskalischen Auswirkungen, Anreizproblematiken und Bewertungen einiger Detailregelungen entstanden und debattiert worden. Nach der ersten Durchführung im Ausgleichsjahr 2020 ist es nun Zeit, erstmalig auf konkrete Ergebnisse einzugehen und dabei verbliebene und neue Probleme offen zu diskutieren.

## Abstract (en)

The new Federal Fiscal Equalization System became effective on 1 January, 2020. While all public budgets in the federal system of Germany were faced with high decreases in revenues caused by the measures to fight the impacts of the COVID-19 pandemic in 2020, a first evaluation shows that the differences to the old system are rather marginal. The focus now lies on the vertical distribution of the value-added tax, whereas for the fiscal capacity equalisation only revenue of the Laender are used as before. In fact, the supplementary federal grants, the federal grants for special needs and very critical new federal grants are still problematic and a strong signal for centralisation.

Mit Beginn des Jahres 2020 ist der neue Finanzausgleich zwischen Bund und Ländern in Kraft getreten. Das erste Jahr der Umsetzung war geprägt durch die COVID-19-Pandemie und ihre Folgen. Hier haben die notwendigen Maßnahmen zur Eindämmung der Pandemie und zum Schutz der Bevölkerungen weltweit die Volkswirtschaften teils dramatisch abgebremst. Auch in Deutschland hat dies in kurzer Zeit zu massiven negativen Folgen sowohl im privaten als auch im öffentlichen Bereich geführt und nicht zuletzt die Einnahmensituation der öffentlichen Haushalte negativ getroffen. Insofern ist 2020 fiskalisch betrachtet ein besonderes Jahr bei der Analyse des neuen Finanzausgleichs. Da jedoch alle öffentlichen Haushalte betroffen sind, lassen sich doch erste wichtige Erkenntnisse gewinnen.

## Das neue System im Kurzüberblick

Da bereits zahlreiche Beiträge das neue System beschrieben haben, werden hier nur die wesentlichen Regelungen für den Finanzausgleich ab 2020 vorgestellt. Nahezu unverändert ist die vertikale und horizontale Zuordnung von Steueraufkommen nach Art. 106 GG sowie Art. 107 Abs. 1 GG. Die Landessteuern werden wie bisher nach dem Prinzip des örtlichen Aufkommens (Art. 107 Abs. 1 Satz 1 GG) den 16 Ländern zugeordnet. Das Aufkommen aus der Einkommen- und Körperschaftsteuer wird dem Prinzip des örtlichen Aufkommens folgend ebenfalls wie bisher einschließlich notwendiger Zerlegungsregeln zugerechnet. Die Umsatzsteuer als dritte Gemeinschaftsteuer nach Art. 106 Abs. 3 GG wird wie bisher auch vertikal aufgrund des im Finanzausgleichsgesetz (FAG) festgeschriebenen vertikalen Verteilungsschlüssels den Ebenen zugeordnet und grundsätzlich nach Bevölkerung verteilt (Art. 107 Abs. 1 Satz 4 GG). Hier setzt die erste Neuerung an. Bei der Verteilung der Länderanteile am Umsatzsteueraufkommen der Länderebene soll nun auch die jeweilige Finanzkraft berücksichtigt werden (Art. 107 Abs. 2 Satz 2 GG).

**Prof. Dr. André W. Heinemann** ist Professor für Bundesstaatliche und regionale Finanzbeziehungen an der Universität Bremen und Leiter der Abteilung Regionalentwicklung und Finanzpolitik des Instituts Arbeit und Wirtschaft (iaw) in Bremen.

Ein Fokus im neuen Finanzausgleichssystem liegt damit nun beim Finanzkraftausgleich (FKA). Hier werden im Rahmen der horizontalen Zuordnung der Umsatzsteueranteile die unterschiedliche Finanzkraft der Länder mitberücksichtigt. Zur Ermittlung der Finanzkraft wird sich wie bisher der Finanzkraftmesszahl (FKM) sowie der Ausgleichsmesszahl (AMZ) bedient. Unter anderem sind das Prämienmodell und Einwohnerwertungen beibehalten worden. Details wie die nun nur noch 33 %ige (bis 2019: 100 %) Anrechnung der Förderabgabe oder die nun 75 %ige (bis 2019: 64 %) Berücksichtigung der gemeindlichen Steuerkraft stellen keine großen Veränderungen des Finanzausgleichssystems, sondern eher kleine fiskalische Anpassungsdetails dar. Eine wesentliche Änderung ist nun die Ermittlung von Abschlägen und Zuschlägen für den FKA im Rahmen der horizontalen Umsatzsteuerzuordnung. Die Höhe der Ab- und Zuschläge ergibt sich nach § 10 Abs. 1 und 2 FAG als 63 %iger Teil[1] des Betrags, um den die FKM die AMZ

1 Damit ist der bisherige dreistufige Tarif mit einer proportionalen und zwei degressiven bzw. progressiven Stufen abgelöst und durch einen durchgängig linearen Tarif ersetzt worden, was eine Vereinfachung des Systems auf dieser Stufe darstellt.

Abbildung 1
**Horizontaler Finanzkraftausgleich und allgemeine Bundesergänzungszuweisungen im Ausgleichsjahr 2020**

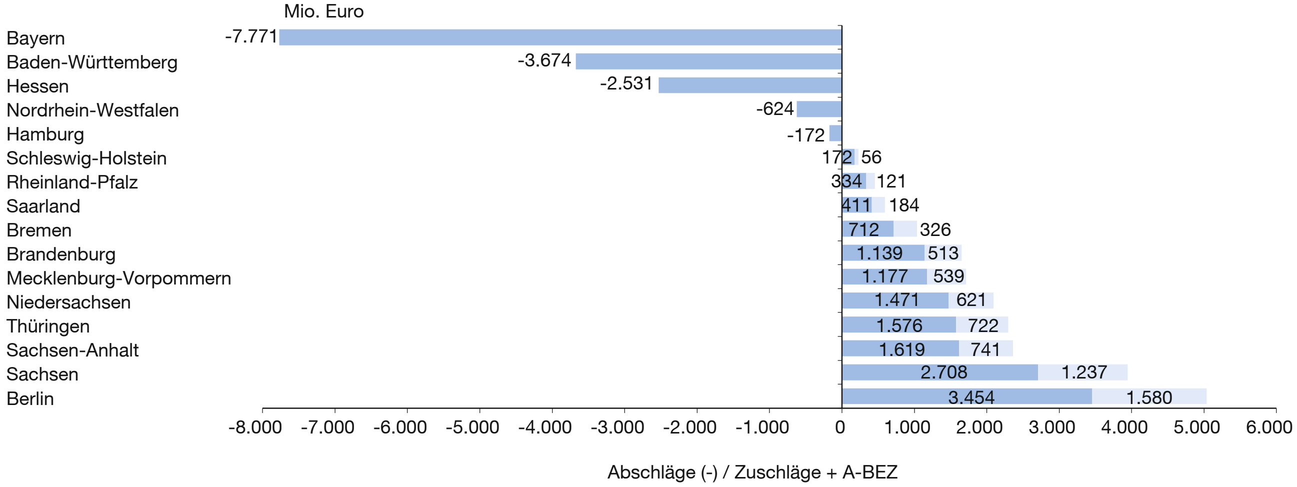

Quelle: Bundesministerium der Finanzen, vorläufige LFA-Abrechnung für 2020; eigene Berechnungen; eigene Darstellung.

übersteigt (Abschlag) bzw. die AMZ die FKM übersteigt (Zuschlag). Damit ist der horizontale FKA abgeschlossen.

Weisen Länder nach FKA noch eine unterdurchschnittliche relative Finanzkraft (Finanzkraftmesszahl + Zuschlag im Verhältnis zur Ausgleichsmesszahl) und gelten somit weiterhin als leistungsschwach, so werden verbliebene Lücken (Fehlbeträge) bis 99,75 % (bis 2019: 99,50 %) zu 80 % (bis 2019: 77,5 %) durch die Vergabe von „Allgemeinen Bundesergänzungszuweisungen" (A-BEZ) durch den Bund aus Bundesmitteln ausgeglichen. Damit ist die allgemeine Anhebung der Finanzausstattung finanzschwacher bzw. leistungsschwacher Länder abgeschlossen.

Der Bund gewährt aus Bundesmitteln im Zuge des sekundären vertikalen Finanzausgleichs wie bisher Sonderbedarfs-Bundesergänzungszuweisungen (S-BEZ) bei Vorliegen von Sonderlasten. Hierzu zählen gemäß § 12 Maßstäbegesetz (MaßStG) die S-BEZ „Kosten politischer Führung" sowie die S-BEZ „Strukturelle Arbeitslosigkeit", die auch bisher gewährt wurden. Neu sind nach Art. 107 Abs. 2 Satz 6 GG Bundesmittel an empfangsberechtigte Länder in Form von BEZ zum „Ausgleich der Gemeindesteuerkraft" sowie BEZ zum „Ausgleich durchschnittsorientierter Forschungsförderung".

## Ab- und Zuschläge im Finanzkraftausgleich

Abbildung 1 zeigt vereinfacht das alte Bild. Weiterhin existiert eine kleinere Gruppe von Ländern, die „Solidarbeiträge" leisten, wobei Bayern mit einem Abschlagsbetrag von über 7,7 Mrd. Euro wie bis 2019 deutlich aus dieser Gruppe hervorragt. Dabei ist jedoch dieser Betrag mit dem Ausgleichsbeitrag von 6,7 Mrd. Euro (2019) für Bayern genauso wenig vergleichbar wie das Ausgleichsvolumen in Höhe von 14,8 Mrd. Euro (2020) gegenüber dem Ausgleichvolumen in Höhe von 11,2 Mio. Euro (2019). Grund hierfür ist die Neugestaltung des FKA, wobei es durch die Verschmelzung von Umsatzsteuervorwegausgleich und Länderfinanzausgleich im engeren Sinne zu einer Abnahme der Ausgleichsstufen gekommen ist. Auch kann formal nicht mehr von Beiträgen gesprochen werden. Im FAG ist von Abschlägen die Rede. Gewissermaßen ist es jeweils ein Verzicht auf einen Anteil am Umsatzsteueraufkommen für ein finanzstarkes Land, der sich bei 100 %iger Verteilung des Länderanteils am Umsatzsteueraufkommen nach Einwohnern auf die 16 Länder ergeben hätte. Da der Ausgleich nun nicht mehr aus bereits in den Länderhaushalten etatisierten Mitteln erfolgt und insofern anders als bis 2019 die Ausgleichsleistungen „nicht mehr in den Landeshaushalten ausgewiesen werden" (BMF, 2021, 21),[2] sondern bereits bei der vertikalen und horizontalen Zuordnung des Länderanteils an der Umsatzsteuer, ist der althergebrachte Begriff „Geberland" nicht mehr zutreffend. Eher kann nun von „Abschlagsland" oder „Verzichtland" gesprochen werden. Zugegebenermaßen weitaus sperrigere Begriffe, weshalb allein schon dadurch die erhoffte positive Wirkung für die öffentliche Diskussion über den Ausgleich zwischen den Ländern im Sinne einer Versachlichung erzeugt wird (Heinemann, 2012, 477). Ein Geben und Nehmen zwischen den Ländern ist weggefallen, was

2 Dies bedeutet auch, dass in den bereinigten Ausgaben der Kassenstatistiken diese Ausgleichsleistungen nicht mehr wie bisher bei den finanzstarken Ländern enthalten sind, wodurch die bereinigten Ausgaben der einzelnen Länder wieder direkt ohne Gefahr der Doppelzählung vergleichbar werden.

Abbildung 2
**Finanzkraft vor Finanzkraftausgleich 2020**

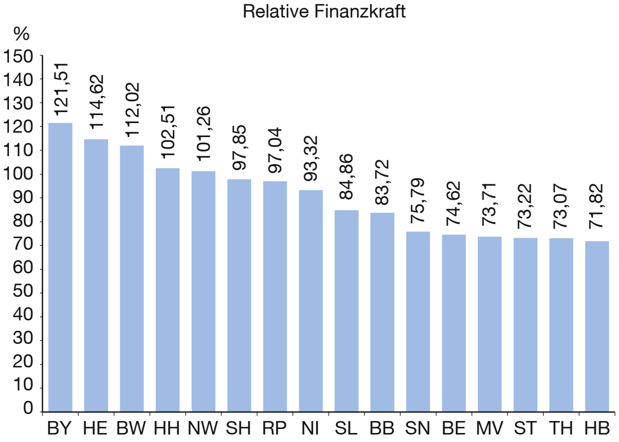

Anmerkungen: Relative Finanzkraft: FKM in % der AMZ. BB = Brandenburg; BE = Berlin, BW = Baden-Württemberg, BY = Bayern, HB = Bremen, HE = Hessen, HH = Hamburg, MV = Mecklenburg-Vorpommern, NI = Niedersachsen, NW = Nordrhein-Westfalen, RP = Rheinland-Pfalz, SH = Schleswig-Holstein, SL = Saarland, SN = Sachsen, ST = Sachsen-Anhalt, TH = Thüringen.

Quelle: Bundesministerium der Finanzen, vorläufige LFA-Abrechnung für 2020; eigene Berechnungen; eigene Darstellung.

Abbildung 3
**Finanzkraft nach FKA und A-BEZ 2020**

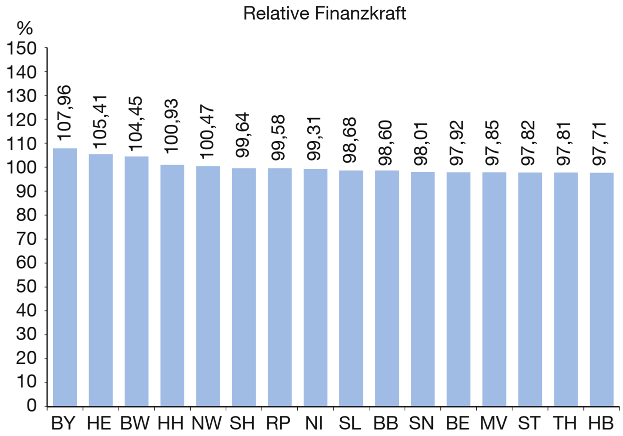

Anmerkungen: Relative Finanzkraft: FKM + A-BEZ in % der AMZ. BB = Brandenburg; BE = Berlin, BW = Baden-Württemberg, BY = Bayern, HB = Bremen, HE = Hessen, HH = Hamburg, MV = Mecklenburg-Vorpommern, NI = Niedersachsen, NW = Nordrhein-Westfalen, RP = Rheinland-Pfalz, SH = Schleswig-Holstein, SL = Saarland, SN = Sachsen, ST = Sachsen-Anhalt, TH = Thüringen.

Quelle: Bundesministerium der Finanzen, vorläufige LFA-Abrechnung für 2020; eigene Berechnungen; eigene Darstellung.

finanzpsychologisch auch für kommende Diskussionen eine Bedeutung haben dürfte.

Durch die Neuregelung des Finanzausgleichs ab 2020 umfasst die Gruppe der finanzstarken Länder nun wieder 60,5 % der Gesamtbevölkerung in Deutschland. Faktisch trägt also wieder eine Mehrheit die Last für Ausgleichsleistungen für eine Minderheit. Dazu beigetragen hat das Land Nordrhein-Westfalen, das nun wieder als finanzstark aufgrund der Neuregelungen bezeichnet wird. Die jeweilige Finanzkraft 2020 vor FKA, ausgedrückt durch die relative Finanzkraft als Verhältnis von Finanzkraftmesszahl (FKM) zu Ausgleichsmesszahl (AMZ), zeigt Abbildung 2.

Der Unterschied zwischen dem finanzstärksten Land (Bayern: 121,51 %) und dem finanzschwächsten Land (Bremen: 71,82 %) vor FKA lag 2020 bei ca. 50 Prozentpunkten. Nach Ausgleich der Finanzkraftunterschiede mithilfe der ab 2020 dafür nun vorgesehenen vertikalen Umsatzsteuerverteilung sowie der sich nach wie vor anschließenden A-BEZ liegt der Unterschied noch bei 10 Prozentpunkten (vgl. Abbildung 3).

Im Ergebnis führten 2020 der FKA (14,8 Mrd. Euro) sowie die Gewährung von A-BEZ (6,64 Mrd. Euro) innerhalb der Gruppe der finanzschwachen Länder dazu, dass abschließend die Differenz zwischen dem finanzschwachen Land mit der höchsten relativen Finanzkraft (Schleswig-Holstein: 99,64 %) und dem finanzschwachen Land mit der geringsten relativen Finanzkraft (Bremen: 97,71 %) keine 2 Prozentpunkte mehr beträgt. Vor FKA betrug die Differenz 2020 mehr als 26 Prozentpunkte (vgl. auch Abbildung 2). Ein erheblicher Anteil des Ausgleichsvolumens, 28,2 % des Finanzkraftausgleichsvolumens und 28,7 % des Volumens der A-BEZ, kommt nach wie vor den Stadtstaaten Berlin und Bremen zugute.

## Neuer FKA – aber mit überwiegend alter Berechnungsmethodik

Durch die Finanzausgleichsregelungen ab 2020 ist der horizontale Ausgleich der unterschiedlichen Finanzkraft der Länder direkt mit der vertikalen Verteilung des Umsatzsteueraufkommen (einschließlich Einfuhrumsatzsteuer) verbunden worden (§§ 4, 5 FAG i.V.m. Art. 107 Abs. 2 Satz 1 und 2 GG). Dabei waren und sind bei der vertikalen Verteilung der Umsatzsteuer nach wie vor folgende Aspekte zu beachten:

- Die vertikale Umsatzsteuerverteilung als grundgesetzlich definierte Gemeinschaftsteuer nach Art. 106 Abs. 3 GG stellt das flexible Element der Finanzverfassung bzw. das „Scharnier“ (Woisin, 2008) zwischen den Finanzausstattungsansprüchen der Bundesebene und der Länderebene dar. Diese vertikale Steueraufkommensverteilung ist nach wie vor anders als die vertikale Verteilung der Aufkommen aus Einkommen- und Körperschaftsteuer einfachgesetzlich im FAG geregelt. Hier hat sich also formal gegenüber den Regelungen bis 2019 nichts geändert. Im Zuge der Reform sind materiell die vertikalen Verteilungsschlüssel neu festgelegt worden, was aber im Zeitablauf immer wieder ein normaler Vorgang und daher üblich ist.

- Auch bis 2019 musste der der Länderebene nach § 1 FAG zustehende Länderanteil am Umsatzsteueraufkommen auf 16 Länder verteilt werden. Dazu wurde auf eine Verteilung nach Bevölkerung nach Art. 107 Abs. 1 Satz 4 1. HS GG (alt) abgestellt: „Der Länderanteil am Aufkommen der Umsatzsteuer steht den einzelnen Ländern nach Maßgabe ihrer Einwohnerzahl zu, (…)". Seit 2020 gilt nun nach Art. 107 Abs. 1 Satz 4 GG: „Der Länderanteil am Aufkommen der Umsatzsteuer steht den einzelnen Ländern, vorbehaltlich der Regelungen nach Absatz 2, nach Maßgabe ihrer Einwohnerzahl zu." Somit hat sich gegenüber den Regelungen bis 2019 auch hier nichts geändert und die Problematik der indirekten Umverteilung ist geblieben.[3]

- Die wesentliche Neuerung im Finanzausgleich unter den Ländern ab 2020 besteht darin, dass der bis 2019 mithilfe von maximal einem Viertel des Länderanteils am Umsatzsteueraufkommen durchgeführte Steuerkraftausgleich[4] weggefallen ist und stattdessen diese verteilungspolitisch motivierte Vorstufe des horizontalen Finanzausgleichs mit dem früheren Länderfinanzausgleich im engeren Sinne verschmolzen wurde. Es bleibt aber dabei, dass der Ausgleich hier wie bisher aus Mitteln erfolgt, die ausschließlich der Länderebene zustehen und insofern der Bund keinerlei Zugriff auf diese Mittel hat. Der Bund kann nur vorab den vertikalen Verteilungsschlüssel ändern, um den Bundesanteil zu erhöhen und so den Länderanteil zu reduzieren. Dazu ist aber stets auch eine Mehrheit im Bundesrat notwendig.

Im Ergebnis hat sich der Bereich des FKA unter den Ländern kaum geändert. Daher soll an dieser Stelle kritisch gefragt werden, ob der neue FKA tatsächlich einen großen formalen Unterschied zum bisherigen System aufweist, wie z. B. Scherf (2020) argumentiert. Auch Bauer, Ragnitz und Rösel (2017, 9) berichten von einer grundlegenden Veränderung der Mechanik der Umverteilung zwischen den Ländern. Tatsächlich sind die grundlegenden Berechnungsvorgaben zur Ermittlung der Finanzkraft der Länderebene sowie der Gemeindeebene einschließlich Prämienregelung und Einwohnergewichtungen beibehalten worden. Daher konstatiert auch das Bundesministerium der Finanzen, dass sowohl die wesentlichen Grundlagen des Finanzausgleichs beibehalten wurden und darüber hinaus „es sich bei dem „Wegfall" des Länderfinanzausgleichs in der Sache darum handelt, dass seine Ergebnisse nunmehr formalrechtlich in die Umsatzsteuerverteilung einbezogen werden" (BMF, 2021, 21).

## Mehr Zentralisierung im bundesstaatlichen Finanzausgleich durch Ausweitung der BEZ

Wie bereits ausgeführt, weist der neue Finanzkraftausgleich gegenüber dem alten Länderfinanzausgleichssystem formal und auch materiell gar nicht so viele Unterschiede auf. Nach wie vor reicht aber offenbar dieser Ausgleich nicht aus, um die Finanzausstattungen finanzschwacher Länder auf ein allgemein akzeptiertes Niveau zu heben. Daher wurde bisher und werden auch im neuen System die noch nach FKA bestehenden Finanzausstattungsdefizite weiter verringert. Dies geschieht mit den dazu vorgesehenen A-BEZ. Diese Mittel stellen tatsächlich Bundesmittel dar, weshalb auf dieser Stufe dann auch die Rolle des Bundes eher zum Tragen kommt als beim horizontalen FKA.

Bereits im alten System standen die BEZ in der Kritik. So wurde auf den eigentlich nur ergänzenden Charakter der BEZ abgestellt (Bickmann und van Deuverden, 2014). Gleichwohl hatte bereits 1986 das Bundesverfassungsgericht (BVerfG) festgestellt, dass sich aus dem ergänzenden Charakter nicht ableiten ließe, dass „diese im Verhältnis zum horizontalen Finanzausgleich nur geringfügig sein dürften" (BVerfGE, 72, 330 (420)). Bei den S-BEZ kommen kritische Fragen hinsichtlich der konkreten und nachvollziehbaren Begründung und Beschreibung von Sonderlasten, der Befristung der Gewährung sowie der zulässigen Änderung der Finanzkraftreihenfolge hinzu.

Die A-BEZ erweitern nur wie bisher den horizontalen FKA auf vertikalem Wege. Altes und neues System eint, dass durch die politische Zielsetzung Länder mit nur sehr geringfügigen Abweichungen zur länderdurchschnittlichen relativen Finanzkraft (bis 2019: > 0,5 Prozentpunkte; ab 2020: > 0,25 Prozentpunkte) nach horizontalem FKA immer noch als leistungsschwach gelten. Wie im alten System wird auch im neuen System durch den FKA eine Leistungsschwäche nicht beseitigt. Dies ist aber letztlich eine politische Vorgabe, denn zusätzliche Bundesmittel zum politisch angemessenen Ausgleich unterschiedlicher Finanzkraft sind nicht unbedingt notwendig. Mit Kenntnis über das Gesamtsystem ist einleuchtend, dass auf die Beteiligung des Bundes an der Finanzausstattungsanhebung mit Bundesmitteln verzichtet werden könnte, wenn nur z. B. im Rahmen

3 „Indirekte Umverteilung", da bereits eine ausschließliche Pro-Kopf-Verteilung mit Blick auf die räumliche Verteilung von Wertschöpfung, Konsum und Umsätzen als Basis des Umsatzsteueraufkommens eine Umverteilungswirkung erzielt. Kitterer (2007, 346) verweist auf das Problem des fehlenden Konsumbezugs der Pro-Kopf-Verteilung des Länderanteils am Umsatzsteueraufkommen und der einhergehenden Umverteilungswirkung, speziell zuungunsten der Stadtstaaten.

4 Zu beachten ist, dass der ursprüngliche Steuerkraftausgleich nur die Ländersteuereinnahmen ohne Umsatzsteueranteile berücksichtigte und somit auf dieser Stufe die Gemeindeebene unberücksichtigt blieb.

der vertikalen Umsatzsteuerverteilung der Länderanteil am Umsatzsteueraufkommen entsprechend erhöht würde. Dabei würde aber eine zielgenaue Anhebung finanzschwacher Länder komplexer, aber auch nicht unmöglich. Warum die Länder den Bund im Grunde unnötiger Weise in den Ausgleich unterschiedlicher Finanzkraft weiterhin einbeziehen, bleibt eine offene Frage. Das Volumen der A-BEZ betrug 6.640 Mio. Euro 2020 gegenüber 4.476 Mio. Euro 2019. Zwar sind aufgrund der pandemiebedingten Steuereinbrüche die beiden Volumina nicht unmittelbar vergleichbar. Aufgrund der Anhebung der Höchstgrenze der Gewährung sowie die Erhöhung des Ausgleichsgrades kann aber dennoch davon ausgegangen werden, dass die Bedeutung der A-BEZ und somit die Rolle des Bundes hier zugenommen hat.

## Besondere Kritik an den S-BEZ

Größerer Kritik sahen sich schon bislang die S-BEZ ausgesetzt. Hier spielte die Frage der Befristung bei den S-BEZ „Kosten politischer Führung“ und bei den S-BEZ „Strukturelle Arbeitslosigkeit“ eine Rolle. Grundsätzlich haben die Gesetzgebenden festgelegt, dass die Vergabe zu befristen und im Regelfall degressiv auszugestalten ist (§ 12 Abs. 3 Satz 1 und 2 MaßStG). In § 12 Abs. 5 Satz 5 MaßstG haben die Gesetzgebenden dann sowohl für die S-BEZ „Kosten politischer Führung“ als auch für die S-BEZ „Strukturelle Arbeitslosigkeit“ die Vorgabe der Befristung mit einer schlichten Formulierung ausgehebelt: „Absatz 3 Satz 1 gilt nicht“. So einfach ist es, Maßstäbe zu setzen und diese umgehend außer Kraft zu setzen.

Die Gewährung der S-BEZ „Kosten politische Führung“ führt zu der Frage, wie effizient die aktuelle Ländergliederung ist, wenn zehn von 16 Ländern die Kosten der Eigenständigkeit nur mit weiteren Bundesmitteln (über 642 Mio. Euro pro Jahr ab 2020; vgl. Tabelle 1) tragen können. Es ist keine Frage, dass bei politisch-administrativen Fixkosten der Eigenständigkeit die Pro-Kopf-Kosten mit ansteigender Bevölkerungszahl sinken und insofern bevölkerungsreiche Länder Kostenvorteile aufweisen. Die Eigenständigkeit bevölkerungsärmerer Länder sollte aber ceteris paribus zu Nutzenvorteilen führen, die die Kostennachteile mindestens kompensieren. In der Tat wäre die Ländergliederung dann effizient. Gerade durch die Gewährung der S-BEZ „Kosten politischer Führung“ kommen Zweifel an einer effizienten Länderstruktur auf und kleine Länder geraten unter politischen und ökonomischen Rechtfertigungsdruck, wenn zusätzliche Kosten für den Bundestaat entstehen. Der Verdacht der Ressourcenineffizienz steht damit im Raum, der dann natürlich mit Verweis auf ausschließlich politische Entscheidungen überlagert werden kann.

Im Zuge der Verhandlungen zur Neuordnung des Finanzausgleichs 2016 sollte das Land Brandenburg pauschal eine im Grunde sachlich unbegründete Erhöhung des Jahresbetrages der S-BEZ „Kosten politischer Führung“ in Höhe von 11 Mio. Euro erhalten (Deutscher Bundestag, 2017). Damit wäre nach der Reform 2005, bei der das Land Rheinland-Pfalz sachlich unbegründet zusätzlich S-BEZ „Kosten politischer Führung“ erhielt (Peffekoven, 2001, 431 f.), ein zweites Mal diese besondere Form der BEZ im Grunde missbräuchlich verwendet worden, um letztlich einen Konsens und ein einstimmig getragenes Verhandlungsergebnis zu erzielen (Heinemann 2017, 212). Aus dieser Situation konnten sich die Gesetzgeber:innen offenbar durch eine auch im Maßstäbegesetz (§ 12 Abs. 3 Satz 3 MaßStG) geforderte Überprüfung befreien. So wurde nach erfolgter Überprüfung festgelegt: „Um die Ergebnisse der Überprüfung der Voraussetzungen für die Vergabe von Sonderbedarfs-Bundesergänzungszuweisungen zum Ausgleich von Sonderlasten leistungsschwacher kleiner Länder aus überdurchschnittlich hohen Kosten politischer Führung umzusetzen, werden die Sonderbedarfs-Bundesergänzungszuweisungen nach § 11 Absatz 4 FAG für die Jahre ab 2020 von insgesamt rund 528 Mio. Euro jährlich auf insgesamt rund 631 Mio. Euro jährlich angehoben. Zudem soll die länderweise Verteilung an die geänderten Einwohnerstrukturen der Ländergemeinschaft angepasst werden“ (Deutscher Bundestag, 2020, 2).

In den genannten 528 Mio. Euro waren die zusätzlichen 11 Mio. Euro für Brandenburg (66,22 Mio. Euro per anno statt 55,22 Mio. Euro per anno.) bereits eingearbeitet (2019: 517 Mio. Euro; vgl. Tabelle 1). Nach der erwähnten Überprüfung mit dem Ergebnis, dass der Gesamtbetrag auf rund 631 Mio. Euro steigen sollte, hätte Brandenburg bei der länderweisen Verteilung nach objektiver Überprüfung auf der Basis ökonometrischer Schätzverfahren 69,674 Mio. Euro per anno erhalten sollen. Allerdings ist den politisch Verantwortlichen dann offenbar wieder eingefallen, dass ja noch die Zusicherung aus 2016 bestand (+11 Mio. Euro). Dies ist dann nachträglich erfolgt (Deutscher Bundestag, 2020a, 1), weshalb ein Jahresbetrag für Brandenburg nun in Höhe von 80,674 Mio. Euro gewährt wird. Offensichtlicher kann die missbräuchliche Nutzung dieser S-BEZ nicht dokumentiert werden.

Die S-BEZ „Strukturelle Arbeitslosigkeit“ sind entstanden im Zuge der Zusammenlegung von Arbeitslosen- und Sozialhilfe für Erwerbsfähige ab 2004. Dabei wurde eine Sonderlast aus dem Umstand abgeleitet, dass damals in ostdeutschen Kommunen eine unterdurchschnittliche Zahl an Sozialhilfeempfänger:innen und gleichzeitig eine überproportional hohe Zahl von Arbeitslosenhilfeempfän ger:innen im Vergleich zu den westdeutschen Kommunen zu berücksichtigen war (Deutscher Bundestag, 2005, 1) und durch die Zusammenlegung von Arbeitslosen- und Sozialhilfe gerade die ostdeutschen Kommunen somit nur unterproportional gegenüber den westdeutschen Kom-

Tabelle 1
**Sonderbedarfs-Bundesergänzungszuweisungen und Bundesergänzungszuweisungen 2020**
in Mio. Euro

| Land | Bisherige S-BEZ | | „Neue" BEZ | | Summe |
|---|---|---|---|---|---|
| | Kosten politischer Führung | Strukturelle Arbeitslosigkeit | Ausgleich Gemeindesteuerkraft | Ausgleich durchschnittsortierte Forschungsförderung | |
| Nordrhein-Westfalen | --- | --- | --- | --- | --- |
| Bayern | --- | --- | --- | --- | --- |
| Baden-Württemberg | --- | --- | --- | --- | --- |
| Niedersachsen | --- | --- | --- | 62,1 | 62,1 |
| Hessen | --- | --- | --- | --- | --- |
| Sachsen | 47,371 (25,565) | 85,492 (160,776) | 433,671 | --- | 566,534 |
| Rheinland-Pfalz | 48,337 (46,016) | --- | --- | 71,1 | 120,037 |
| Sachsen-Anhalt | 70,993 (52,663) | 50,116 (94,248) | 230,108 | 12,4 | 363,617 |
| Schleswig-Holstein | 66,308 (53,174) | --- | --- | 7,2 | 73,508 |
| Thüringen | 71,432 (55,731) | 47,168 (88,704) | 247,945 | 18,9 | 385,445 |
| Brandenburg | 80,674 (55,220) | 50,920 (95,760) | 25,043 | 3,4 | 160,037 |
| Mecklenburg-Vorpommern | 71,959 (61,335) | 34,304 (64,512) | 191,252 | 4,9 | 302,415 |
| Saarland | 66,309 (63,400) | --- | 23,067 | 3,5 | 92,876 |
| Berlin | 58,671 (43,460) | --- | --- | --- | 58,671 |
| Hamburg | --- | --- | --- | --- | --- |
| Bremen | 60,332 (60,332) | --- | --- | --- | 60,322 |
| Summe | 642,386 (516,916) | 268,000 (504,000) | 1.151,09 | 184,1 | 2.245,57 |

Anmerkungen: In Klammern Werte für das Ausgleichsjahr 2019.

Quellen: Bundesministerium der Finanzen (2021), Monatsbericht des BMF, März 2021, 25-26; Bundesministerium der Finanzen (2020), Finanzbericht 2021, Oktober 2020, 179; § 11 Abs. 3 und 4 FAG; eigene Berechnungen entsprechend § 11 Abs. 5 FAG.

munen profitierten. Diese damals neuen S-BEZ „Strukturelle Arbeitslosigkeit" waren bis 2009 befristet und sollten 2008 erstmalig überprüft werden. Bereits 2006 wurde die Befristung um ein Jahr auf 2010 verlängert und seit 2009 ist die grundsätzliche Befristung entfallen. Mit insgesamt 268 Mio. Euro (vgl. Tabelle 1) spielen sie aber quantitativ längst nicht mehr die Rolle wie noch 2005 (1.000 Mio. Euro). Gleichwohl weisen Bickmann und van Deuverden (2014, 681) darauf hin, dass eine Finanzschwäche eigentlich entweder durch den horizontalen Finanzausgleich oder durch A-BEZ ausgeglichen werden sollte.

## Sonderbedarf oder doch kein Sonderbedarf? – Neuartige BEZ ab 2020

Auf der Grundlage des reformierten Art. 107 Abs. 2 GG können nun Zuweisungen des Bundes „unabhängig von den Maßstäben nach den Sätzen 1 bis 3 auch solchen leistungsschwachen Ländern gewährt werden, deren Gemeinden (Gemeindeverbände) eine besonders geringe Steuerkraft aufweisen (Gemeindesteuerkraftzuweisungen), sowie außerdem solchen leistungsschwachen Ländern, deren Anteile an den Fördermitteln nach Artikel 91b ihre Einwohneranteile unterschreiten" (Art. 107 Abs. 2 Satz 6 GG).

Für Scherf (2020, 603) zeigt sich die Tendenz der Vertikalisierung im Finanzausgleich auch bei den BEZ für Sonderbedarfe und führt als Beispiel sogleich die neuartigen BEZ „Gemeindesteuerkraft" auf. Zudem weist Scherf darauf hin, dass im Falle der zweiten neuartigen BEZ zum Ausgleich durchschnittsorientierter Forschungsförderung überhaupt kein Sonderbedarf vorliege. Bei beiden neuartigen BEZ sollte ergänzt werden: Sonderbedarfe haben die Gesetzgeber:innen offenbar auch gar nicht als Voraussetzung vorgesehen. Ansonsten hätten diese BEZ in § 12 MaßstG (S-BEZ) aufgenommen werden können. Sind sie aber nicht. Vielmehr sind diese neuartigen BEZ in § 11 MaßstG (Zuweisungen nach Artikel 107 Absatz 2 Satz 6 des Grundgesetzes) zu finden. Dort ist von Sonderlasten oder -bedarfen keine Rede.

Unter anderem kritisiert Scherf (2020), dass für die Finanzkraft beide Ebenen eines Landes konsolidiert, also das „gemeinsame Potenzial der Länder und Gemeinden zur Finanzierung öffentlicher Aufgaben und Ausgaben" (Scherf, 2020, 603), betrachtet werden müsse. So aber wird nun systematisch die Länderebene von der Gemeindeebene getrennt und die interne Finanzverteilstruktur ist auf dieser Stufe nicht mehr maßgeblich. Dadurch kommt es, wie auch bereits bei Scherf (2020) vorhergesagt, zu Situationen, bei denen Länder aufgrund unterdurchschnittlicher kommunaler Finanzkraft[5] zusätzliche BEZ erhalten, obwohl diese Länder in der Gesamtschau die gleiche Gesamtfinanzkraft oder sogar eine höhere Gesamtfinanzkraft aufweisen wie nicht zuweisungsberechtigte Länder aufgrund ihrer nichtunterdurchschnittlichen kommunalen Finanzkraft. So weist Bremen eine relative Finanzkraft (Länder- und Gemein-

5 Der Fehlbetrag, der zu 80 % des Durchschnitts besteht, wird zu 53,5 % aufgefüllt, § 11 Abs. 5 FAG. Berücksichtigt werden die kommunalen Steuereinnahmen nach § 8 Abs. 1 und 2 FAG, also ohne Herabsetzung auf 75 % nach § 8 Abs. 3 FAG.

deebene) nach horizontalem FKA und A-BEZ von 97,7 % 2020 auf und das Saarland verfügt nach horizontalem FKA und A-BEZ über eine relative Finanzkraft von 98,68 % (vgl. Abbildung 3). Obwohl das Saarland also bereits eine höhere Finanzkraft hatte, erhielt das Land 2020 noch weitere rund 23 Mio. Euro zusätzlich nur aufgrund der unterdurchschnittlichen kommunalen Steuerkraft (vgl. auch Tabelle 1).

Zusätzlich können die BEZ „Gemeindesteuerkraft“ bewirken, dass sich die Finanzkraftreihenfolge verändert.[6] Darauf hatte Scherf (2020) ebenfalls hingewiesen. So zeigen die Ergebnisse für 2020, dass etwa Mecklenburg-Vorpommern nach horizontalem FKA und A-BEZ eine relative Finanzkraft von 97,85 % erreicht, während Rheinland-Pfalz auf 99,58 % kommt. Nach Gewährung von über 191 Mio. Euro in Form von BEZ „Gemeindesteuerkraft“ (vgl. Tabelle 1) erreicht Mecklenburg-Vorpommern dann 100,55 % und überholt Rheinland-Pfalz. Zwar hat das BVerfG eine Veränderung der Finanzkraftreihenfolge durch S-BEZ als zeitweise tolerierbar eingestuft, dabei aber deutliche Hinweise gegeben:

> „Berücksichtigt der Gesetzgeber bei der Gewährung von Bundesergänzungszuweisungen dagegen Sonderlasten einzelner Länder, ist nicht ausgeschlossen, situationsabhängig und insoweit zeitlich begrenzt Zuweisungen auch solchen Ländern zu gewähren, deren Finanzkraft nach Durchführung des Länderfinanzausgleichs den Länderdurchschnitt erreicht oder überschritten hat (BVerfGE 72, 330, 404 f.). Demzufolge können Bundesergänzungszuweisungen, die gerade der Berücksichtigung von Sonderbedarfen dienen, zeitweise zu Veränderungen der Finanzkraftreihenfolge führen; das Nivellierungsverbot greift insoweit nicht. Allerdings müssen für die Berücksichtigung von Sonderlasten außergewöhnliche Gegebenheiten vorliegen, die einer besonderen, den Ausnahmecharakter ausweisenden Begründungspflicht unterliegen. Im Regelfall darf die Gewährung von Bundesergänzungszuweisungen auch bei der Berücksichtigung von Sonderlasten nicht dazu führen, daß die Finanzkraft des begünstigten Landes die durchschnittliche Finanzkraft der Länder nach dem horizontalen Finanzausgleich übersteigt. Darüber hinaus ist der Gesetzgeber aus dem föderativen Gleichbehandlungsgebot auch verpflichtet, die Sonderlasten zu benennen und zu begründen (BVerfGE 72, 330, [405 f.])." (BVerfGE 101, 158, 234 f.).

Fraglich bleibt, ob es sich entsprechend des Wortlautes des Maßstäbegesetzes aber überhaupt um S-BEZ handelt. Vielmehr orientieren sich die BEZ „Gemeindesteuerkraft“ nach § 12 MaßStG, wenngleich „formal unabhängig von den Maßstäben des Finanzkraftausgleichs“ (Scherf, 2020, 603), eher an den Maßstäben für die A-BEZ, wobei allerdings nur die kommunale Ebene und diese abweichend zum horizontalen FKA mit 100 % berücksichtigt wird. Auch muss kritisch darauf hingewiesen werden, dass es letztlich zu einer doppelten Berücksichtigung der kommunalen Steuerkraft im bundesstaatlichen Finanzausgleich kommt, da die kommunale Ebene bzw. Steuerkraft bereits im horizontalen FKA berücksichtigt wird (Heinemann, 2017, 211).

Zuletzt wurden im Ausgleichsjahr 2020 insgesamt 184,1 Mio. Euro durch den Bund in Form der neuartigen BEZ „Ausgleich durchschnittsorientierte Forschungsförderung“ vergeben. Diese Gewährung kann letztlich nicht mit einem „Sonderbedarf“ in Verbindung gebracht werden, da er schlicht nicht vorliegt (Scherf, 2020, 604). Hier wird grundsätzlich nur eine finanzielle Differenz zwischen dem eigenen Nettozufluss aus den Mitteln der Forschungsförderung nach Art. 91b GG und dem durchschnittlichen Nettozufluss aus der Forschungsförderung teilkompensiert (35 % der zu 95 % des Durchschnitts vorhandenen Lücke). In anderen Worten: Erhält ein Land nur einen unterdurchschnittlichen Nettozufluss im Rahmen der Forschungsförderung, so fließen dem Landeshaushalt dann doch weitere Bundesmittel zu. Für Niedersachsen kamen 2020 über 62 Mio. Euro zusätzlich für den Landeshaushalt zusammen. Den höchsten Betrag erhielt Rheinland-Pfalz mit 71,1 Mio. Euro (vgl. Tabelle 1).

Da es sich bei S-BEZ letztendlich um ungebundene Haushaltsmittel handelt, „die zur ergänzenden Deckung des allgemeinen Finanzbedarfs gewährt werden“ (Deutscher Bundestag, 2006, 17), stehen empfangsberechtigten Ländern die Mittel zur allgemeinen Verfügung. Unterdurchschnittliche Nettozuflüsse aus der Forschungsförderung nach Art. 91b GG erhöhen also prinzipiell den Haushaltsspielraum eines Landes auch für Ausgaben jenseits der Wissenschafts- und Forschungsaktivitäten (z. B. Innere Sicherheit, Verkehr, Umweltschutz).

## Abschließende Bemerkungen

Der neue Finanzausgleich wurde erstmalig 2020 durchgeführt. Dabei hat sich am Gesamtbild wenig geändert. „Geberländer“ und „Nehmerländer“ existieren zwar nicht mehr, dafür kann nun von „Abschlagsländern“ und „Zuschlagsländern“ gesprochen werden. Die in den vergangenen Jahren vorgebrachte Kritik, dass durch die Vertikalisierung des Ausgleichs zwischen den Ländern die Solidarität zwischen ihnen aufgekündigt sei (Renzsch, 2017), wird nicht geteilt. Immerhin verzichtet z. B. Bayern 2020 auf über 7,7 Mrd. Euro im Rahmen des horizontalen FKA, was durchaus als solidarischer Beitrag zur Unterstützung finanzschwacher

6 Dies gilt auch grundsätzlich für die S-BEZ „Kosten politischer Führung“, für die S-BEZ „Strukturelle Arbeitslosigkeit“ sowie für die BEZ „Ausgleich durchschnittsorientierte Forschungsförderung“.

Länder betrachtet werden kann. Neu ist, dass die „horizontale Solidarität" nun weniger sichtbar ist (Lenk und Glinka, 2017, 507). Dafür ist der nach wie vor hohe Nivellierungsgrad durch FKA und A-BEZ weiterhin erkennbar.

Insgesamt dürften in der weiteren Diskussion über einen angemessenen, bedarfsorientierten und solidarischen Finanzausgleich in Deutschland und das Verhältnis der Bundesebene zur Länderebene (Stichwort: schleichende Zentralisierung) die BEZ eher Gegenstand kritischer und kontrovers geführter wissenschaftlicher und politischer Auseinandersetzungen über die Begründung der Ansprüche, die Sinnhaftigkeit im Föderalstaat sowie über die Vergabemodalitäten sein als der neue FKA. Aber auch hier sind natürlich einige Probleme verblieben.
